# Elder Orphans Hiding in Plain Sight: A Growing Vulnerable Population

**DOI:** 10.1155/2016/4723250

**Published:** 2016-10-23

**Authors:** Maria T. Carney, Janice Fujiwara, Brian E. Emmert, Tara A. Liberman, Barbara Paris

**Affiliations:** ^1^Long Island Jewish Medical Center, Northwell Health, Hofstra Northwell School of Medicine, New Hyde Park, NY 11040, USA; ^2^Maimonides Medical Center, Icahn School of Medicine at Mount Sinai, Brooklyn, NY 11219, USA

## Abstract

Adults are increasingly aging alone with multiple chronic diseases and are geographically distant from family or friends. It is challenging for clinicians to identify these individuals, often struggling with managing the growing difficulties and the complexities involved in delivering care to this population. Clinicians often may not recognize or know how to address the needs that these patients have in managing their own health. While many such patients function well at baseline, the slightest insult can initiate a cascade of avoidable negative events. We have resurrected the term* elder orphan* to describe individuals living alone with little to no support system. Using public data sets, including the US Census and University of Michigan's Health and Retirement Study, we estimated the prevalence of adults 65 years and older to be around 22%. Thus, in this paper, we strive to describe and quantify this growing vulnerable population and offer practical approaches to identify and develop care plans that are consistent with each person's goals of care. The complex medical and psychosocial issues for elder orphans significantly impact the individual person, communities, and health-care expenditures. We hope to encourage professionals across disciplines to work cooperatively to screen elders and implement policies to prevent* elder orphans* from hiding in plain sight.

## 1. Introduction

It is common for physicians who provide care to older adults to encounter an* elder orphan* in their office, hospital, or an emergency room, but they do not recognize them as such or identify the risks related to this.* We define elder orphans as aged, community-dwelling individuals who are socially and/or physically isolated, without an available known family member or designated surrogate or caregiver*. This demographic, those aging alone with limited support, is expected to increase as the United States population continues to age and people live in the community with more chronic illnesses. Recent national media reports have also brought attention to this growing problem [1–3]. Thus, we strive to raise awareness of the concept of aging alone without an available caregiver and introduce the term* elder orphan* to more clearly define this vulnerable population and identify these individuals as high risk in an effort to call to action health-care providers, government agencies, and general public to address their needs and minimize preventable illness. We also provide guidance on how to screen and care for an individual who may be at risk for being an* elder orphan*.

Below, two case scenarios are described which underscore the concepts and risks involved with* elder orphans*. These cases highlight the crucial need to identify members of this population in order to prevent medical crises.

**Case 1 (Ms. H. M.).**

Ms. H. M. is a 92-year-old widow living in her home with her 65-year-old son with cerebral palsy, who is dependent upon her. She has managed to live at home with little help for many years. Over the past few months, however, she noticed that her function is declining; she is becoming unable to drive or even do many household chores. Moreover, because of a growing lethargy, she is finding it more difficult to even cook and clean. A fiercely independent woman, she has attempted to hire aides, but she promptly lets them go because of difficulty supervising them.

After meeting with a social worker while still able to make decisions and through an introduction to legal guidance in the community, a plan of action was determined for both her and her son, as well as the beginnings of preparations of what would happen with her son if anything were to happen to her. A distant but willing family member was reconnected and helped support the plan created. Through identification of her and her son's risk to be “orphaned” and the creation of a care-giving plan and identification of a health-care proxy or surrogate decision-maker, the likelihood of medical catastrophe for this elder orphan and her son (who will eventually inherit elder orphan status) has decreased significantly.

**Case 2 (Mr. H. B.).**

Mr. H. B. is a 72-year-old man living alone in his apartment in Long Island, New York. He was admitted to a Palliative Care Unit for complex medical, social, and wound care after a failed suicide attempt, having slit his wrists with a razor. Upon admission, it was found that Mr. H. B. was never married and was childless and his closest relative was residing in California, thus uninvolved in his care. Mr. H. B.'s relative had little knowledge of his condition. Once wound care was complete, finding placement for Mr. H. B. was difficult, as he was not healthy enough to travel to California to be near his only relative, nor was he psychologically or medically well enough to be discharged home alone. With no known caregiver identified, after a several-week stay in the hospital, he was eventually relocated to a skilled nursing facility for further wound care with a long-term plan to be relocated near his only relative in California.

The term* elder orphan* was utilized on rounds with Case 2 to highlight the vulnerability of individuals with limited to no support in the community whose abilities are being challenged and risk of losing independence is significant. This particular case led to much discussion and academic interest because of an additional perceived increase in individuals being seen at the hospital who lack care-giving and decision-making support by spouse, partner, family, or community.

For patients like Ms. H. M. and Mr. H. B., we utilize the term* elder orphan*. It is imperative that the medical and social community become more familiar with this term as it highlights a population aging alone without a caregiver and with significant barriers to care. Furthermore, the term* elder orphan* when utilized properly creates an important notification to health-care providers that care-giving needs are lacking and are an important aspect to treatment. Moreover, we expect the prevalence of those aging alone and those who are at risk of being* elder orphans* to continue to increase as individuals are living longer, with multiple chronic diseases, alone, and geographically distanced from other family members. Thus, in this paper, our goals are threefold: to evaluate the terms synonymous with aging alone or “elder orphan” use in literature, identify the prevalence of being at risk to be* elder orphans* and the risks facing this population, and provide guidance when faced with caring for an* elder orphan*.

## 2. Methods

### 2.1. Literature Search

A literature search was undertaken to examine the use of the term* elder orphan or any term synonymous with age, isolated, and/or alone*. To better characterize this vulnerable population and identify clinical correlates for risk factors, four databases were searched: PubMed, Google Scholar, Health Reference, and CINAHL. Reviews of police and emergency management department programs, U.S. Census data, and the North Shore-LIJ Health System social work database were also conducted to assist in terminology use for vulnerable adults. The search terms utilized included* elder orphan*,* unbefriended elder, patients without surrogates, vulnerable elderly, social isolation, loneliness, childless unmarried, frail elderly, lone elders*, and* aging alone* as shown in the following list (synonyms encountered in reviewing the medical literature on social isolation in older adults).

*Similar terms encountered while searching “elder orphan” are the following:*

 Aging alone.
 Elder orphan.
 Frail elderly.
 Patients without surrogates.
 Social isolation.
 Unbefriended elder.
 Vulnerable elderly.

A total of 56 publications were identified and reviewed from international medical, legal, and lay press sources dating back approximately 35 years (Table 1).

### 2.2. Prevalence

Estimates of the prevalence of* elder orphans* living in the United States were determined by using previously published, valid, and publicly accessible national surveys. Initially, we conducted an analysis of U.S. Census data. We then turned our attention to the Health and Retirement Study (HRS) [4]. The HRS is sponsored by the National Institute on Aging (Grant number NIA U01AG009740) and is conducted by the University of Michigan. It surveyed a representative sample of over 22,000 people in the United States aged 65 years and older about aspects of their personal life and family. We recoded and parsed the data so as to examine marital status, number of children, number of children in contact, number of children in close proximity, number of siblings, and number of siblings in close proximity of the subject. From this analysis, we devised a spectrum of categories which, by definition, can lead to aged, isolated, alone status. We then extrapolated the prevalence (in percent of the population) for each tier in the spectrum, using the numbers derived from the HRS, and then further estimated the prevalence of at-risk elder orphans in the general population.

## 3. Results

### 3.1. Literature Search

#### 3.1.1. Use of Term

Through the literature search efforts the term* elder orphan* was found to be first designated by Kunerth [5] in 2003 and Sherer [6] in 2004 in the lay press, and then it resurfaced in a state nursing journal in 2005 in a didactic article by Varner [7] to describe a growing subset of the geriatric population that requires special consideration. Since then, the term has been dormant, and as a consequence pertinent concepts related to the* elder orphan*, such as the estimated prevalence of and risks associated with being an* elder orphan*, are not well documented in the medical literature.

We propose that this term be resurrected out of dormancy in order to highlight a need to intervene for social support with a goal of minimizing adverse medical events and frailty.* Elder orphans* are a unique subset of the aging population, as their inclusion in this category is often due to circumstance rather than choice. As independent individuals, they have functioned sufficiently well on their own and thus do not actively plan for their medical future. As they age and decline, they realize, often too late, that they can no longer complete many of the tasks that they were previously able to do. Stemming from this inability,* elder orphans* may no longer access the care that they need, and acute, possibly preventable, medical events occur that can easily lead to hospitalization. These events often incur significant costs to the health system and undue suffering to the patient. By raising awareness for this group of aging adults by referencing them with a benevolent and informative title such as* elder orphan*, we hope this group will get more attention by the medical and social community. Advanced planning and consciousness will be raised for these individuals, and with coordination between medical and community organizations they can be directed to appropriate services before their function declines, facilitating maintenance of quality of life in their own communities for as long as possible.

The term* elder orphan* raises clear awareness to medical providers of the vulnerability of the individual and the importance of managing the patient's care comprehensively and multidisciplinarily.

### 3.2. Risks

#### 3.2.1. Social Support

In addition to the likelihood of not receiving adequate care, being at risk for* elder orphan* status (aged, community-dwelling individuals who are socially and/or physically isolated, without an available known family member or designated surrogate or caregiver) can have a series of adverse biopsychosocial consequences on an individual. Low social support has been linked to both poor physical and psychological health and an increased risk of mortality for the elderly population [8, 9]. Moreover, decreased social interaction that can stem from this lack of support is correlated with low affect and arousal [10], poor cognitive and social skills [11], and altered neurophysiological functioning [12].

#### 3.2.2. Isolation and Loneliness

Isolation and loneliness are distinct in that isolation is the objective state of having minimal contact with others, whereas loneliness is the subjective feeling of being socially alone and isolated. Both of these states have been identified as risk factors for physical and cognitive decline.

Perissinotto et al. [13] completed a longitudinal cohort study of 1604 subjects and found that among those who are 60 years of age and older loneliness was a predictor of both functional decline (in areas including mobility, climbing, upper extremity tasks, and activities of daily living) and death. Additionally, Sorkin et al. [14] found that greater levels of loneliness and lower levels of emotional support and companionship were correlated with an increased risk of coronary disease.

Social isolation has been shown to be a risk factor for medical complications and mortality. Wenger et al. [15] studied working class individuals and found that social isolation is correlated with advancing age; being male and single; living alone; and having no children. These researchers also found an association between social isolation and retirement migration (moving to a new area upon retirement), poor health, restricted mobility, admission to institutional care, low morale, poor rehabilitation, and mental illness.

In a study of 271 community-dwelling elderly women, Thompson and Heller [16] found that both subjectively and objectively isolated women had poorer psychological wellbeing than the population mean. Moreover, those who were objectively isolated comorbidly exhibited poorer functional health. Finally, Udell et al. [17] found that in an international outpatient population with atherothrombosis living alone was associated with both increased cardiovascular death and four-year mortality, a trend which was found to grow stronger as the population aged.

#### 3.2.3. Marriage and Children

Being married provides advantages for medical care and support for patients. In an analysis of childless elderly patients discharged from a hospital, marital status was found to be a major determinant of the level of support the patient received after discharge. Although childless, married individuals tended to rely solely on each other and thus were more socially isolated, they were resourceful in using long-term accumulation of social resources to meet their needs [18].

Childlessness is an important risk factor for social isolation. Many studies have shown that childless adults often do have support networks, usually consisting of relatives, friends, and neighbors. However, these networks are less likely to provide the long-term commitment and comparable high level of support that children offer [19, 20]. Interestingly, evidence is inconclusive regarding the long-term difference between childless older adults and elders with children. Although the childless elderly appear to score lower on measures of objective social support, another evidence suggests that their psychological wellbeing does not significantly differ from older adults with children [21]. It is important to note that gender was a mediating factor; Zhang and Hayward found that childless men had higher rates of loneliness and depression than childless women.

An interesting concept regarding childlessness in the elderly arises when considering parents who outlive their children. The loss of a child can cause severe psychosocial stress on an individual, especially when the child dies as a result of disease. Parents may attribute the death as resulting from their actions or perceived inactions and, as part of their bereavement process, socially isolate themselves [22]. If the parent is older or single, this isolation can have devastating consequences on his or her health and welfare.

Another trend that may further impact adults outliving their children is described in the American Medical Association 2012 study that found that the current generation may be the first to encounter parents outliving their children. This is attributed to childhood obesity which in turn increases rates on hypertension, diabetes, stroke, and osteoarthritis upon reaching middle age. The University of Michigan's Joyce Lee found that people born between 1966 and 1985 became obese at much faster rates than previous generations [23].

### 3.3. Prevalence

According to 2010 U.S. Census data, nearly 19 percent of women aged 40 to 44 years have no children, as compared to about 10 percent in 1980 [24]. Furthermore, in 2009, almost one third of Americans aged 45–63 years are single, a 50 percent increase from 22% in 1980 [25]. There are no signs of this trend reversing. While being a parent or spouse does not guarantee care in old age, the bulk of America's elderly are cared for primarily by their spouses and children [26].

Limited data exists to measure the prevalence of this population. HRS data was used to estimate aging alone with limited support using marital status, having children, having siblings, or having children or siblings not in contact or not within 10 miles (existing HRS criteria and surrogate conditions deemed by authors as possible local care-giving involvement ability) (Table 2). Based on data from the HRS, we estimate that the prevalence of being at high risk for* elder orphan* status is to be as high as 22.6%. Fortunately, we found that individuals who are most likely already* elder orphans*, by definition, are just a small percentage of the population (Table 2).

## 4. Discussion

We define elder orphans as aged, community-dwelling individuals who are socially and/or physically isolated and have no known family member or designated surrogate available to them. Both the safety and the independence of this demographic are threatened. With the high prevalence of individuals aging alone and the clear risks associated, it is crucial that the medical and social community become aware of this pressing issue. Moreover, the medical community must actively screen and take steps to care for individuals who fall into this demographic; consider the following lists: Questions to Screen for Risk for* Elder Orphan* Status and Ten-Step Guide to Caring for an* Elder Orphan*.

*Questions to Screen for Risk for Elder Orphan Status*

- Do you have a spouse or significant other?
- Do you have children? Are they nearby?
- Do you have family members or friends that help you cope with life challenges?
- Do you have someone to help you make medical decisions?
- Do you have someone to help with bills, financial decisions?
- Do you have a health-care proxy or any advance directives?
- Who is the person you would call upon in an emergency or crisis situation?
- Do you have a home health aide to help with personal care such as bathing, dressing, and other activities of daily living?

*Ten-Step Guide to Caring for an Elder Orphan*

*(1) Identify All Medical Issues*. This may involve speaking with the patient's known providers and other personal contacts, telephoning pharmacies, and accessing old charts, laboratory work, and imaging studies. Consider asking the following:

- Have you fallen in the past 6 months?
- Do you have 3 or more chronic illnesses?
- Do you take 5 or more medications?
- Have you been hospitalized in the past 3 months?

*(2) Identify Cognitive and Functional Abilities*. Use of cognitive, depression, and functional assessment tools (e.g., Mini-Cog Assessment, Geriatric Depression Scale, Activities of Daily Living, and Instrumental Activities of Daily Living assessments) may be particularly helpful with the patient's care assessment and plans for discharge [27–30]. Consider asking the following:

- Do you need help with bathing, dressing, shopping, and paying bills?
- Do you feel sad?
- Are you lonely?

*(3) Obtain Detailed Social Support Information*. It is important to call any possible contacts that may be beneficial in identifying care for* elder orphans*. This may include out-of-town family, friends, neighbors, physicians, and significant others. Furthermore, all resources and benefits available for the patient need to be identified. A social worker can assist with gathering some of the information. Consider asking the following:

- Who could help you in a crisis?
- Do you have a long-term care policy?
- Are you a veteran in the military?

*(4) Create a Manageable and Realistic Treatment Plan*. Individuals without support need to have treatment plans that can be achieved. 

*(5) Utilize Service Delivery to Home*. For example, utilize home care, pharmacy, and food delivery services.

*(6) Make Safety and Injury Prevention a Priority; Address Safety and Injury Issues.* Consider asking the following:

- Have you fallen?
- Do you have a gun in your home?
- Are you driving? Did you experience any accidents? Do you wear your seatbelt regularly? Have you gotten lost while driving?

*(7) Address Goals of Care and Advance Directives*. By focusing on health-care proxy and living will, future resuscitation, mechanical ventilation, treatment, hospitalization, and even funeral and burial arrangement wishes may elicit support systems that exist. Consider asking the following:

- Do you have a health-care proxy or durable power of attorney for healthcare?
- Do you have a living will?
- Do you have a will for your belongings, property? Who has helped you with these?
- Have you discussed future treatment, hospitalization, burial wishes, or arrangements with anyone or made future plans?

*(8) Understand Privacy Issues (HIPAA)*. Health-care workers must be cognizant of privacy laws while understanding that the intent of reaching out to support systems is to assist in medical care and health advocacy. Health professionals must fully document that the purpose of outreach is for the safety and health of the individual, and, in so doing, privacy laws are respected but do not form a barrier to coordination of care.

*(9) Assess Decision-Making Capacity and Involve the Individual as Much as Possible*. Assess whether the person has the ability to make specific decisions, as capacity is valid solely on a case-by-case basis and based on a specific issue being decided on. Although a person may be failing in some cognitive abilities, it does not necessarily mean that they lack the ability to make certain health-care decisions [31].

*(10) Determine If Guardianship Is Needed, and If So, Seek It*. A guardianship is a legal relationship created when a person or institution is named in a will or assigned by the court to take care of incompetent adults. Consider contacting hospital legal or social work departments.

In Questions to Screen for Risk for* Elder Orphan* Status we outline key screening questions that can help health-care providers identify individuals at risk of being* elder orphans*. These suggested questions can be self-administered or easily incorporated into other assessments completed by office assistants to help identify individuals at risk. Further studies on the effectiveness of these questions as a screening tool are needed. As shown in the Ten-Step Guide to Caring for an* Elder Orphan*, we have developed these 10-steps to assist providers in sorting through the complex physical and psychosocial issues that elder orphans face. We offer practical approaches to developing a multidisciplinary, holistic approach and care plan for these individuals to address a growing public health need.

Identifying these individuals prior to loss of function or admission into acute care facilities will help to expedite appropriate medical care, avert negative outcomes, and reduce the burden on the health system. Early identification of these at-risk individuals allows for care plans that can better meet the needs of the elder orphan.

We suggest the term* elder orphan* as a benevolent identifier for a group of individuals who find themselves in this difficult situation. We hope to incite awareness and action in the medical and social community to assist these older adults in society who are unable to complete instrumental activities of daily life and have no available caregivers, as well as those who are at risk of isolation and lacking support. Although other terms have been used to describe individuals who fall within the category of vulnerable older adults (e.g., the unbefriended elderly who are alone and lack decision-making ability), they have the potential to inadvertently stigmatize these individuals and often fall within legal realm. Thus, we resurrect the use of* elder orphan* as a benevolent and medical alternative to a more broad population of individuals who are alone and unsupported.

The purpose of the term,* elder orphan*, is for use in health-care environment to highlight vulnerability and attract attention to the need for a care-giving and medical decision-making plan. What is not known is how use of this term might negatively impact the individual. It is the authors' hope that use of the term will lead to allocation of more resources for the individual. Further studies should investigate the impact of the use of the term on care on the individual and potential for unintended negative consequences such as stigmatization.

A limitation to our estimation of prevalence is that with the available data the physical and cognitive health of the subjects' relatives and friends is unknown, such as in Case 1, Ms. H. M. Future studies should analyze these variables in order to offer a more accurate prevalence. Moreover, as the data is based on marital status, it does not provide measurement of individuals with significant or domestic partners that are involved caregivers. More detailed analyses are needed to more accurately measure an at-risk* elder orphan* population.

The expected future increase in the number of individuals without support from children and/or spouses/partners, combined with a population that is living longer, poses an enormous challenge to both the health-care system and the community. Thus, further studies are needed to elucidate the exact prevalence of this population, the needs of this group, and the resources currently available to them. Moreover, a critical view of the risks of being an* elder orphan* must be delineated in order to more adequately prepare for and minimize them. In these future studies, care must be taken to examine the number, health status, and relationship of the subjects' existing family members, as well as their marital status and health-care advocates.

In addition, the services needed for this population should be scrutinized. This at-risk group requires access to a host of services in order to help them thrive independently in the community. Those identified as* elder orphans* should be educated about advanced directives and creating a plan of care far in advance of needing acute care. More importantly, they should receive assistance as needed and as available to implement and achieve a plan of care. A few simple measures can help stem catastrophe, and some possible resources needed for this population are the following:

- Community based aging resource centers and adult day care centers (community access to social services and senior organizations with a goal of preventing avoidable hospital admissions).
- Community multidisciplinary teams to care for patients with medical, functional, social, and safety needs.
- Public-private partnerships to help vulnerable populations, linking health-care teams with community and government agencies (e.g., social services, adult protective services, and senior agencies).

Based on our clinical experience and a literature review, we propose ten steps physicians and other providers should take into account to identify and help address the medical and psychosocial needs of elder orphans in the community, as outlined in Ten-Step Guide to Caring for an* Elder Orphan*.

## 5. Conclusion

The* elder orphan* population is an increasingly prevalent and at-risk demographic living precariously in the community. They often go unrecognized by health-care providers and the community alike, silently living in danger of medical crises. The medical, public health, and general community need to become more aware of these individuals in order to protect and advocate for them. Our proposed screening questions in Questions to Screen for Risk for* Elder Orphan* Status and ten-step guide in Ten-Step Guide to Caring for an* Elder Orphan* can help when faced with caring for an older adult with no one. Further action is vital but steps, as outlined, could begin to address this growing population, identify the needs, raise awareness in order to mobilize public health and community resources, and prioritize development of care-giving and decision-making plans, so that these individuals are no longer hiding in plain sight.

## Figures and Tables

**Table 1:** The table shows the results of a literature search regarding previous work completed on elder orphans. Search terms of “elder orphan,” “unbefriended elderly,” “patients without surrogates,” and “vulnerable elderly” were used in Google Scholar, PubMed, CINAHL, and Health Reference databases.

Author	Year	Title	Comments
*Elder orphan*			

Soniat B. & Pollack M.	1994	“Elderly Orphans with Alzheimer's Disease: Non-Traditional Support Systems”	Describes differences between a functional family system and an informal support network providing assistance to an “elderly orphan”

Sherer R. A.	2004	“Who Will Care for Elder Orphans?”	Geriatric Times article describing a growing population and a bill to expand medical training programs

*Unbefriended elderly*			

Gillick M. R.	1994	“Medical Decision-Making for the Un-Befriended Nursing Home Resident”	Reviews pathways of addressing decision-making in nursing home residents without decision-making capacity or surrogates, such as use of ethics committees

Freeman I. C.	1995	“One More Faulty Solution Is Novelty without Progress: A Reply to “Medical Decision-Making for the Un-Befriended Nursing Home Resident””	Refutes the idea that a simple, less cumbersome process is needed for nursing home residents without decision-making capacity or surrogates

Teaster P. B.	2002	“The Wards of Public Guardians: Voices of the Unbefriended”	Through qualitative data collection and analysis, this study explores the interaction between adult public guardianship wards and their public guardians, including ward satisfaction and perceptions, guardian responsiveness, and investigation of the guardian-ward relationship

Karp N. & Wood E.	2004	“Incapacitated and Alone: Healthcare Decision-Making for Unbefriended Older People”	Synopsis of a 2003 American Bar Association Commission on Law and Aging report. This report found limited existing studies on the unbefriended elderly; however, available estimated data was compelling enough for the Commission to issue recommendations to ensure the ethical treatment of the unbefriended elderly

Pope T. M. & Sellers T.	2011	“Legal Briefing: The Unbefriended: Making Healthcare Decisions for Patients without Surrogates”	Overview of legal developments in medical decision-making for the unbefriended elderly, including an outline of the problems involved in this type of medical decision-making and a selection of potential solutions

Johnstone M.-J.	2014	“Caring about the Unbefriended Elderly”	A presentation of the shortcomings of interventions for the elderly when the individual is an unbefriended elder and a call for nurses to identify and advocate for this vulnerable group of patients

*Patients without surrogates*			

Meier D. E.	1997	“Voiceless and Vulnerable: Dementia Patients without Surrogates in an Era of Capitation”	Describes how the growth of a dementia population will add to the dilemma of difficulties of complex health-care decision-making and the increase in fiscal pressures by health-care environments while advocating for caution of undertreatment of the most vulnerable

Miller T. E., Coleman C. H., & Cugliari A. M.	1997	“Treatment Decisions for Patients without Surrogates: Rethinking Policies for a Vulnerable Population”	Provides an outline of the laws and policies surrounding decision-making for incapacitated individuals without surrogates, discusses the substitutes used by state governments that exist, and provides a model for healthcare to assist these special needs populations

Chichin E. R.	2004	“Ethics and the Elderly”	Addresses health-care decision-making with elderly and uses case examples to explore situations

Crampton A.	2004	“The Importance of Adult Guardianship for Social Work Practice”	This article reviews the guardianship decision-making process and how social workers can have a role in promoting social justice through this process

Frank C.	2005	“Surrogate Decision-Making for “Friendless” Patients”	This article reviews surrogate decision-making and explores how and why alternative means of substituted decision-making must be found

Quinn M. J.	2005	*Guardianships of Adults*	Describes the history and institution of guardianship, provides alternatives to guardianship, explains the criteria for guardianship, and describes the court process and court monitoring of guardianships for community health and social service practitioners

Siegel M. D.	2006	“Alone at Life's End: Trying to Protect the Autonomy of Patients without Surrogates or Decision-Making Capacity”	Comments on White D. B. article in Critical Care Medicine 2006 exploring decision-making on critically ill individuals without surrogates and highlights the need for more attention on this vulnerable group

Castillo L. S., Williams B. A., Hooper S. M., & others	2011	“Lost in Translation: The Unintended Consequences of Advance Directive Law on Clinical Care”	Describes potential negative clinical impact of advance directive laws on all patients and particularly on vulnerable populations

Pope T. M.	2013	“Making Medical Decisions for Patients without Surrogates”	Describes this marginalized, vulnerable population and addresses the need to focus attention on prevention of being without a surrogate

*Vulnerable elderly*			

Morris J. N. & Sherwood S.	1983	“Informal Support Resources for Vulnerable Elderly Persons: Can They Be Counted on, Why Do They Work?”	The issue of informal support system resiliency is analyzed for approximately 700 vulnerable elderly persons in a variety of communities

Auerbach M., Taylor M., & Marosy J.	1985	“Home Care Challenge: Care of the Vulnerable Elderly”	Early describer of provision of care to an at-risk population in the home

Davidson B.	1985	“Vulnerable Elderly in Acute Care Settings: A Developing Model”	Early describer of creation of a new model of care focusing on an at-risk population

Thomas B. L.	1994	“Research Considerations: Guardianship and the Vulnerable Elderly”	Challenges inherent assumptions in guardianship protocol and calls for more research into risks and benefits of guardianship assignment and decisions

Shapiro E.	1998	“Market Forces and Vulnerable Elderly People: Who Cares?”	An editorial calling for the need of regulations and quality control to protect vulnerable elders from cost containment initiatives

Billipp S. H.	2001	“The Psychosocial Impact of Interactive Computer Use within a Vulnerable Elderly Population: A Report on a Randomized Prospective Trial in a Home Health Care Setting”	Compared weekly nurse visits with nurse and interactive computer use and found that interactive computer use could be an added and more beneficial resource for isolated, vulnerable older adults in the community to minimize

Grundy E.	2006	“Ageing and Vulnerable Elderly People: European Perspectives”	Describes the processes and circumstances that create vulnerability among older people, specifically to a very poor quality of life or an untimely or degrading death. Policy initiatives describe that aim to reduce vulnerability by focusing on each part of the dynamic process that creates vulnerability, namely, ensuring that people reach later life with “reserve,” reducing the challenges they face in later life, and providing adequate compensatory supports

Rollins J. N.	2006	“Office-Based Intervention Improves Vulnerable Elderly Care”	Describes quality improvement initiative focusing on vulnerable older adults

Cumbler E., Carter J., & Kutner J.	2008	“Failure at the Transition of Care: Challenges in the Discharge of the Vulnerable Elderly Patient”	Describes the challenges facing individuals with limited social support for smooth transitions of care

Franzini L. & Dyer C. B.	2008	“Healthcare Costs and Utilization of Vulnerable Elderly People Reported to Adult Protective Services for Self-Neglect”	Describes a study that demonstrates the same or potentially less cost to a health-care system by referral of self-neglectors to APS and a geriatric health-care team, demonstrates that costs are not higher, and theorizes that costs may be even less because preventive efforts are initiated to provide support before a medical crisis occurs

Morley J. E.	2008	“Caring for the Vulnerable Elderly: Are Available Quality Indicators Appropriate?”	Explores and questions appropriateness of ACOVE-3 guidelines

Rosenberg J. A.	2009	“Poverty, Guardianship, and the Vulnerable Elderly: Human Narrative and Statistical Patterns in a Snapshot of Adult Guardianship Cases in New York City”	Explores guardianship, explains demographics such as gender, race, socioeconomic, and housing characteristic association with risk for need of guardianship (e.g., women living alone are more likely to be in need), and uses cases to describe

Day M. R., Bantry-White E., & Glavin P.	2010	“Protection of Vulnerable Adults: An Interdisciplinary Workshop”	Provided an interdisciplinary shared learning experience for the students to prepare them for their critical role in safeguarding vulnerable adults. The aim of the workshop was to increase knowledge, awareness, and understanding of roles and responsibilities and critical practice problems in the prevention and management of elder abuse and self-neglect

Harrington P., Preston J., Savattone D., & Volk-Craft B.	2010	“Establishing Healthcare-Related Legal Options for the Vulnerable Patient”	Explores legal options for individuals with no surrogate and having no decision-making capacity

Shaffer S. L. & Day H. D.	2010	“Systematic Outpatient Screening for the Elderly: Care of the Vulnerable Elderly Practice Improvement Module to Assess Resident Care of Older Adults”	Describes a practice improvement project to screen for vulnerable elderly geriatric needs including surrogate decision-maker; a potential model to identify those aging alone

van Hout H. P., Jansen A. P., van Marwijk H. W., & others	2010	“Prevention of Adverse Health Trajectories in a Vulnerable Elderly Population through Nurse Home Visits”	Studied the effect of a preventative home care nursing visit program but failed to decrease hospital admissions

Glasper A.	2013	“Improving Health Care for the Vulnerable Elderly in Society”	Explores how a department of health can improve healthcare for the vulnerable elderly

**Table 2:** The table shows the prevalence of those at risk of becoming an elder orphan based on 2010 data. Prevalence was calculated by dividing the sum of the total individuals in the “unmarried, with children, but not in contact” tier and the “unmarried, without children” tier (the two biggest risk factors for becoming an *elder orphan*) by the total of respondents to the health and retirement study [4].

Risk description	Number	Percent (out of 22,034 respondents)
Unmarried, with children, but not in contact	3,903	17.7%
Unmarried, with children, but they are not in contact, and there are no siblings within 10 miles	3,738	17.0%
Unmarried, with children, but children live further than 10 miles away	3,106	14.1%
Unmarried, with children, but not within 10 miles, and there are no siblings within 10 miles	48	0.2%
Unmarried, without children	1,071	4.9%
Unmarried without children or siblings	141	0.6%
Totally unmarried, without children, or unmarried with children, not in contact	4,974	22.6%

Total prevalence of at-risk individuals = (unmarried, with children, not in contact) + (unmarried without children) = (3,903 + 1,071)/22,034 = 22.6%.

## References

[1] Kleinfeld N. R. (2015). *The Lonely Death of George Bell*.

[2] Span P. (2015). *The Last ‘Friend’ You May Ever Need*.

[3] Tribune Media Wire Service Elderly man with cancer calls 911 because he has no food. http://fox6now.com/2015/05/13/elderly-man-with-cancer-calls-911-because-he-has-no-food/.

[4] Health and Retirement Study (2010). *(Family Data) Public Use Dataset*.

[5] Kunerth J. (2003). *Seniors Outliving Family, Becoming Elder Orphans*.

[6] Sherer R. (2004). Who will care for elder orphans. *Geriatric Times*.

[7] Varner J. (2005). The elder orphans: who are they?. *The Alabama Nurse*.

[8] Blazer D. G. (1982). Social support and mortality in an elderly community population. *American Journal of Epidemiology*.

[9] Seeman T. E. (1996). Social ties and health: the benefits of social integration. *Annals of Epidemiology*.

[10] Larson R. (1985). Being alone verus being with people: disengagement in the daily experience of older adults. *Journals of Gerontology*.

[11] Dolen L. S., Bearison D. J. (1982). Social interaction and social cognition in aging. A contextual analysis. *Human Development*.

[12] Arnetz B. B., Theorell T., Levi L., Kallner A., Eneroth P. (1983). An experimental study of social isolation of elderly people: psychoendocrine and metabolic effects. *Psychosomatic Medicine*.

[13] Perissinotto C. M., Cenzer I. S., Covinsky K. E. (2012). Loneliness in older persons: a predictor of functional decline and death. *Archives of Internal Medicine*.

[14] Sorkin D., Rook K. S., Lu J. L. (2002). Loneliness, lack of emotional support, lack of companionship, and the likelihood of having a heart condition in an elderly sample. *Annals of Behavioral Medicine*.

[15] Wenger G. C., Davies R., Shahtahmasebi S., Scott A. (1996). Social isolation and loneliness in old age: review and model refinement. *Ageing and Society*.

[16] Thompson M. G., Heller K. (1990). Facets of support related to well-being: quantitative social isolation and perceived family support in a sample of elderly women. *Psychology and aging*.

[17] Udell J. A., Steg P. G., Scirica B. M. (2012). Living alone and cardiovascular risk in outpatients at risk of or with atherothrombosis. *Archives of Internal Medicine*.

[18] Johnson C. L., Catalano D. J. (1981). Childless elderly and their family supports. *The Gerontologist*.

[19] Beckman L. J., Houser B. B. (1982). The consequences of childlessness on the social-psychological well-being of older women. *Journals of Gerontology*.

[20] Choi N. G. (1994). Patterns and determinants of social service utilization: comparison of the childless elderly and elderly parents living with or apart from their children. *The Gerontologist*.

[21] Zhang Z., Hayward M. D. (2001). Childlessness and the psychological well-being of older persons. *Journals of Gerontology B: Psychological Sciences and Social Sciences*.

[22] Mahgoub N., Lantz M. (2006). When older adults suffer the loss of a child. *Psychiatric Annals*.

[23] Lee J. M., Pilli S., Gebremariam A. (2010). Getting heavier, younger: trajectories of obesity over the life course. *International Journal of Obesity*.

[24] Monte L. M., Ellis R. R. (2014). Fertility of Women in the United States: 2012. *Commerce USDo*.

[25] Lin I.-F., Brown S. L. (2012). Unmarried boomers confront old age: a national portrait. *Gerontologist*.

[26] American Association of Retired P, United S. Aging/Parents & Adult Children Together, A/PACT, Washington, D.C, USA, Federal Trade Commission, AARP, 1999

[27] Borson S., Scanlan J. M., Chen P., Ganguli M. (2003). The Mini-Cog as a screen for dementia: validation in a population-based sample. *Journal of the American Geriatrics Society*.

[28] Sheikh J. I., Yesavage J. A. (1986). Geriatric Depression Scale (GDS): recent evidence and development of a shorter version. *Clinical Gerontologist*.

[29] Katz S. (1983). Assessing self-maintenance: activities of daily living, mobility, and instrumental activities of daily living. *Journal of the American Geriatrics Society*.

[30] Lawton M. P., Brody E. M. (1969). Assessment of older people: self-maintaining and instrumental activities of daily living. *The Gerontologist*.

[31] Appelbaum P. S., Grisso T. (1988). Assessing patients' capacities to consent to treatment. *The New England Journal of Medicine*.

